# Optic disc hemorrhage in nonglaucomatous eyes: A cross-sectional study with average 8-year follow-up

**DOI:** 10.1371/journal.pone.0237796

**Published:** 2020-08-17

**Authors:** Yeo-Yang Koh, Chi-Chun Lai, Henry S. L. Chen, Ling Yeung, Wan-Chen Ku, Lan-Hsin Chuang

**Affiliations:** 1 Department of Ophthalmology, Chang Gung Memorial Hospital, Keelung, Taiwan; 2 Department of Ophthalmology, Chang Gung Memorial Hospital, Linkou, Taiwan; 3 College of Medicine, Chang Gung University, Taoyuan, Taiwan; LV Prasad Eye Institute, INDIA

## Abstract

**Purpose:**

To characterize changes in the retinal nerve fiber layer (RNFL) and peripapillary vessel density (VD) at the site of disc hemorrhage (DH) in nonglaucomatous eyes.

**Materials and methods:**

This retrospective cross-sectional study included nonglaucomatous eyes diagnosed with unilateral DH. The change of DH was recorded using disc photography. Both anatomical data and functional visual field (VF) data were collected using optical coherence tomography angiography and Humphrey VF examination.

**Results:**

Sixteen patients were included with average follow-up duration of 95 months. Almost half of DH episodes was initially presented at the inferotemporal area of the optic disc. Pigment formation at the previous DH site after resolution was noted in 12.5% of eyes. Sectoral radial peripapillary VD at the DH site was significantly lower in DH eyes than in the control group; however, the sectoral RNFL thickness at the DH site was not significantly decreased. Progression of the VF defect corresponding to the DH site was found in 81.3% of eyes despite regular use of antiglaucoma agents. The mean change in the VF mean deviation was –0.64 dB/year in DH eyes.

**Conclusion:**

During long follow-up periods, decreased peripapillary VD at the DH site and progression of the VF defect corresponding to the DH site were detected in nonglaucomatous eyes. Retinal pigmentation with an RNFL defect is a clue for DH, although RNFL showed no significant change. Antiglaucoma treatment may not prevent the deterioration of visual function.

## Introduction

Optic disc hemorrhage (DH) was initially considered a precursor for a glaucomatous change because DH is detected before the diagnosis or progression of glaucoma.[1] In addition to the evolution of DH as a diagnostic tool, it is known as a risk factor for glaucoma.[2, 3] Medeiros et al. demonstrated the visual field progress after DH in glaucoma patients recruited from the Diagnostic Innovations in Glaucoma Study.[3] DH anatomically develops in association with notching, progressive changes in the optic disc rim,[4] localized retinal nerve fiber layer (RNFL) defects,[5] and focal visual field progression[6] in glaucoma as a functional change.

In the literature, there is no consensus related to the long-term effect of DH on nonglaucomatous eyes. In clinical practice, we have observed that some eyes with DH present neither the history nor the signs of glaucoma before the diagnosis of DH. Prior studies have reported the detection of a small percentage of DH in eyes without glaucoma.[7–9] However, most of the studies have investigated DH in glaucomatous eyes. Limited studies have conducted the structural and functional evaluation of DH in nonglaucomatous eyes.

The new advent of optical coherence tomography angiography (OCT-A) has provided us a new perspective on various ocular diseases. This emerging technique yields both qualitative and quantitative microvascular data and allows evaluation of disc perfusion. Park et al. used disc angiography to identify vessel-filling defects and delayed filling of vessels of the optic nerve head in glaucomatous eyes with DH.[10] Park et al. concluded that in glaucoma with DH, choroidal microvascular changes are associated with progressive RNFL thinning.[11] However, to date, no OCT-A study has investigated microcirculation changes in nonglaucomatous eyes with DH.

This study characterized the change in RNFL and peripapillary vessel density (VD) at the site of DH in nonglaucomatous eyes.

## Materials and methods

This hospital-based cross-sectional study was conducted at Chang Gung Memorial hospital, Keelung branch, Taiwan. This study was approved by the Institutional Review Board of Chang Gung Memorial Hospital and followed the tenets of the Declaration of Helsinki. Patients diagnosed with optic DH as per characteristic clinical features were included. Subjects with glaucoma were excluded initially. Included patients also met the following exclusion criteria: a cup-disc ratio of >0.5; a history of any retinal disease, including diabetic and hypertensive retinopathy; a history of eye trauma or surgery with the exception of uncomplicated cataract surgery; optic nerve disease; fellow eye with a diagnosis of glaucoma; and a history of systemic or neurologic diseases that might affect the VF. The fellow eyes that met the aforementioned exclusion criteria were included as the control group.

Demographic data, systemic diseases, and treatment regimens were recorded. Each patient had a comprehensive ophthalmic examination, including a review of medical history, demographic data, visual acuity measurement, slit-lamp biomicroscopy, fundoscopic examination, and axial length measurement. Serial disc photographs and charts were evaluated during the follow-up period. DHs were described as flame-shaped or splinter hemorrhages perpendicular to the optic disc margin.[12] Recurrent DH including DH at the same location or a different location was defined as eyes with more than one DH during follow-up. Retinal pigmentation formation along the disc margin at the prior DH site was recorded.

Optic disc and macular VD were analyzed using OCT-A (AngioVue; Optovue, Inc., Fremont, CA, USA). The device performs an A-scan at a rate of 70,000 scans per second in 3 s by using an 840-nm superluminescent diode. Two consecutive B-scans (M-B frames), each containing 304 A-scan, were captured by the RTVue system. A macular volumetric scan centered on the fovea with an area of 3 × 3 mm^2^ was performed. The OCT-A imaging system used split-spectrum amplitude-decorrelation angiography to extract OCT-A information. For macular VD, software of the OCT angiogram segmented full-thickness retinal scans into “superficial” and “deep” inner retinal vascular plexuses. The superficial inner retina included the vasculature in the area between the RNFL and ganglion cell layer, whereas the deep inner retina showed the vascular plexuses between the border of the inner plexiform layer (IPL) and inner nuclear layer (INL) and the border of the INL and outer plexiform layer.

For optic disc vessel density, in an optic nerve head image with an area of 4.5 × 4.5 mm, the peripapillary VD was calculated from the “radial peripapillary capillary segment” extending from the inner limiting membrane (ILM) to the posterior border of the RNFL. The peripapillary region is divided into eight sectors, each of 45 degrees, as per the Garway-Heath map, and peripapillary VD in each sector is calculated.[13] We recorded the change of peripapillary VD, sectoral VD at the DH site; subsequently, we compared data with the corresponding sector in the fellow eye of the patient.

Ganglion cell complex (GCC) and circumpapillary RNFL thickness were measured using OCT-A. The GCC thickness was calculated between the ILM and the outer border of the IPL in an area of 7 × 7 mm, whereas circumpapillary RNFL was measured in a circle of 3.45-mm diameter from the center of the optic disc.[14]

To monitor the visual function, standard automated perimetry (Humphrey Field Analyzer II; Carl Zeiss Meditec, Dublin, CA, USA) was performed. A visual field test with low reliability, including ≥33% fixation losses, ≥10% false positives, ≥10% false negatives, and OCT-A with low signal strength index (<50), was excluded from this study. Baseline visual field examination was performed during the detection of DH, and follow-up visual field examinations were performed annually. Visual field progression was described as the presence of two or more points that declined by at least 10 dB from the mean baseline values of these points between the baseline and final visual field examinations with the VF defect extending from the sectoral area of the DH location. The progression was calculated by the decreased mean deviation (MD) slope (dB/year) during follow-up examinations.

For statistical analysis, demographic data and clinical data, including sectoral RNFL and VD at the DH site, were compared between the lesion eye and fellow eye by using independent Student’s *t* test. All statistical analyses were performed using SPSS software, version 20.0 (SPSS, Inc., Chicago, IL, USA). P values less than 0.05 were considered to be statistically significant.

## Results

Of 19 patients eligible for this study, three eyes were excluded because of inadequate OCT-A imaging; finally, 16 eyes were included in our study. All included patients had unilateral DH, and their fellow eyes were allocated to the control group. The mean age of patients was 68.9 years, and patients were predominantly women (83.3%). The average follow-up duration was 7.9 years (95 months), whereas the mean baseline intraocular pressure (IOP) was 14.4 mmHg (Table 1).

**Table 1:** Characteristics of nonglaucomatous patients with DH.

Variables	Values
**Age** (years, mean ± SD)	68.4 ± 9.56
**Sex (male/female)**	3/13
**Self-reported history of diabetes** (n, %)	7 (43.8%)
**Self-reported history of hypertension** (n, %)	4 (25.0%)
**Use of anti-glaucoma agents** (n, %)	11 (68.8%)
**Peripapillary atrophy area** (n, %)	
Yes	11 (68.8%)
No	5 (31.2%)
**Axial length** (mm, mean ± SD)	24.1 ± 1.62
**Type of DH** (n,%)	
Peripapillary hemorrhage	16 (100%)
Cup hemorrhage	0
**Shape of DH** (n, %)	
Flame-shaped hemorrhage	13 (81.3%)
Dot hemorrhage	3 (18.7%)
**Location of initial DH** (n, %)	
Inferotemporal	7 (43.7%)
Superotemporal	5 (31.2%)
Inferonasal	2 (12.5%)
Superonasal	1 (6.3%)
Temporal	1 (6.3%)
**Location of recurrent DH** (n, %)	
Inferotemporal	4 (57.1%)
Superotemporal	2 (28.6%)
Inferonasal	1 (14.3%)
**Progression of visual field defect** (n, %)	13 (81.3%)
**Recurrence of DH** (n, %)	5 (31.3%)
**Pigment formation after DH** (n, %)	2 (12.5%)
**Follow-up duration** (months, mean ± SD)	94.9 ± 43.51

DH: disc hemorrhage

**Fig 1:**
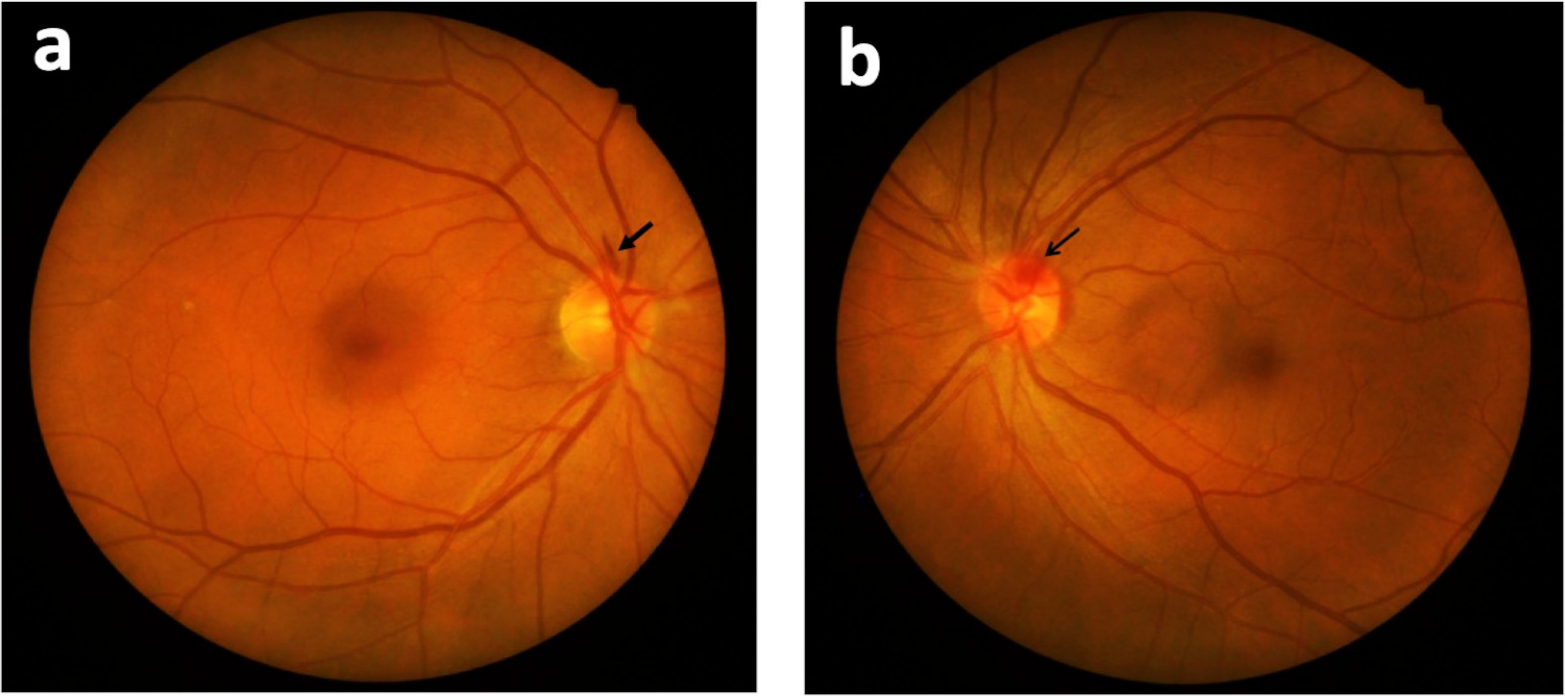
Two types of peripapillary DH. (a) A 64-year-old woman with flame-shaped peripapillary hemorrhage (arrow). (b) A 59-year-old woman with dot peripapillary hemorrhage (thin arrow).

**Fig 2:**
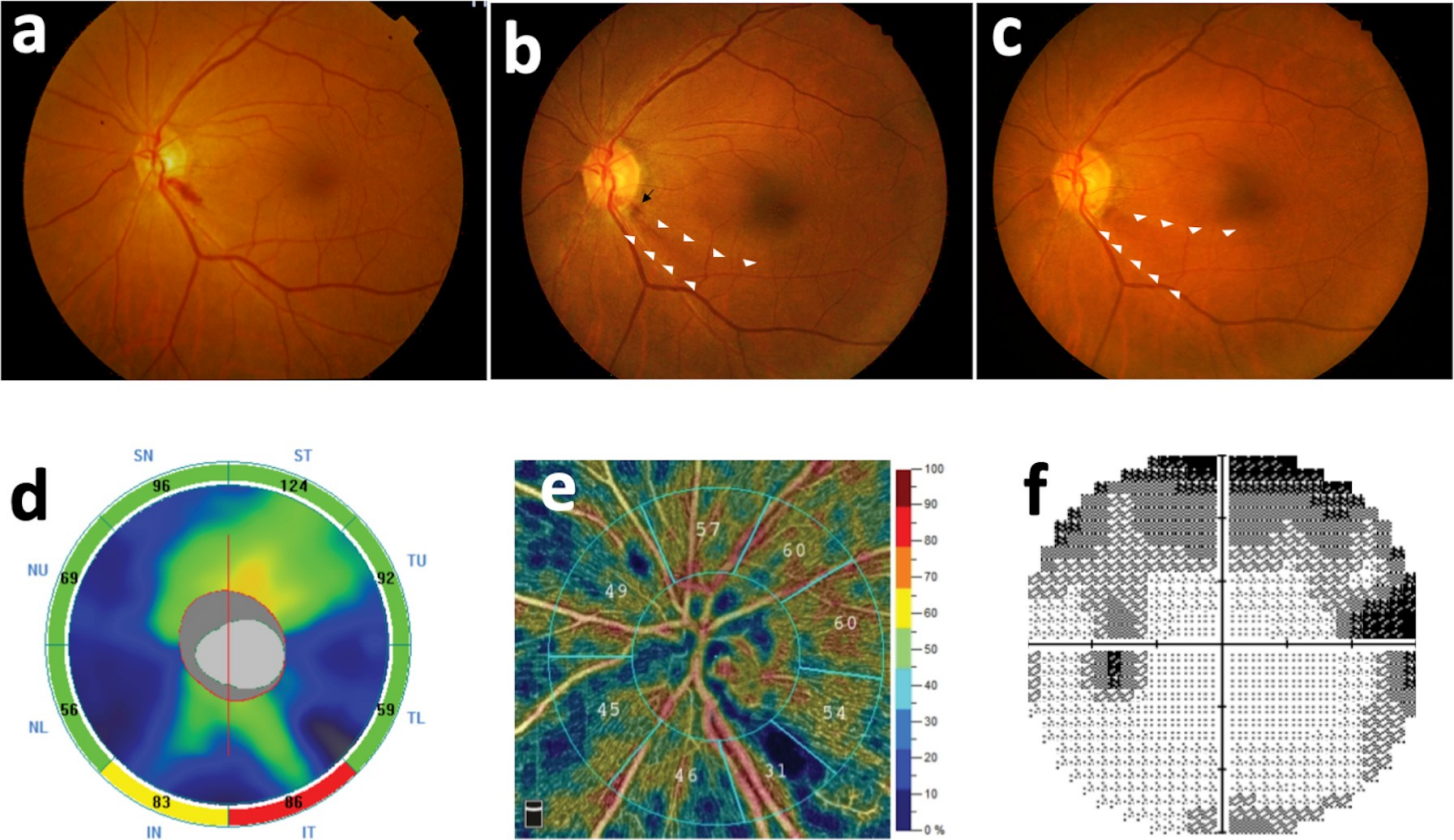
A 71-year-old woman with peripapillary pigment formation subsequent to resolution of DH after a 6-year follow-up. (a) This patient initially presented with peripapillary DH at a 5 o’clock position. (b) 2 years later, pigment formation (arrow) with an inferotemporal RNFL defect (white arrowheads) developed at the prior site after the resolution of DH. (c) 4 years later, the pigmentation remained and the RNFL defect progressed (white arrowheads). (d) & (e) Optical coherence tomography and angiography revealed RNFL thinning and decreased VD in the inferotemporal area corresponding to findings on a color fundus photo. (f) Humphrey visual field examination disclosed superior arcuate scotoma.

**Fig 3:**
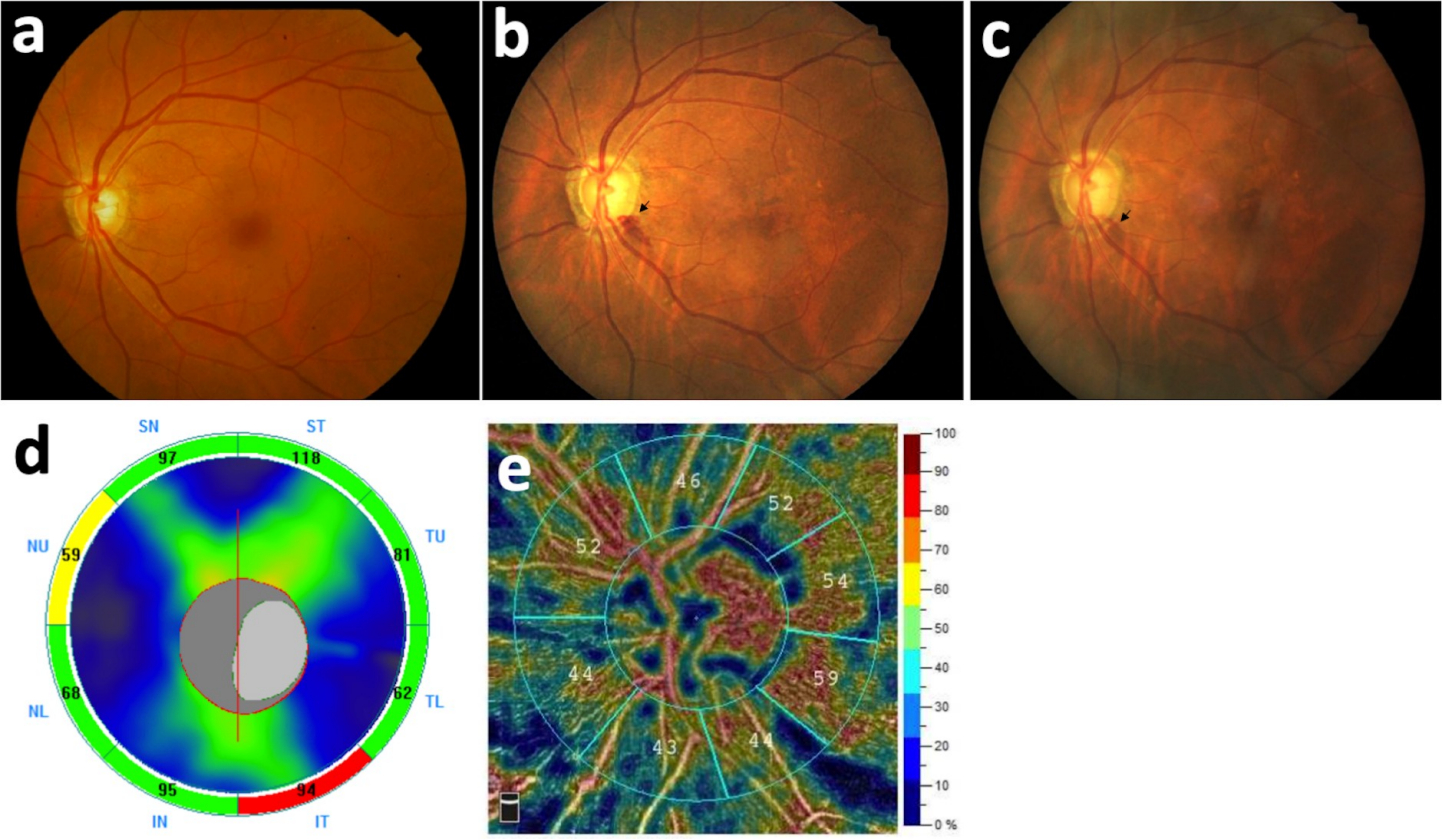
A 67-year-old woman with peripapillary pigmentation following resolution of DH after a 2-year follow-up. (a) Disc photography before DH. (b) Peripapillary DH (arrow) developed at a 5 o’clock position. (c) 2 years later, pigment formation (arrow) at prior site after the resolution of DH. (d) & (e) In final visit, optical coherence tomography and angiography revealed RNFL thinning and decreased VD in the superotemporal area corresponding to findings on a color fundus photo.

All included eyes initially presented with peripapillary DH (none of them was a cup DH); 81.3% DH were flame-shaped, whereas 18.7% were dot hemorrhages (Fig 1). DH occurred most commonly in the inferotemporal area (43.7%), followed by superotemporal, inferonasal, superonasal, and temporal areas. Particularly, we found subsequent retinal hyperpigmentation developing at the resolved DH site in two eyes after 2 years and 4 years from the onset of DH, respectively. Both the eyes were found to have a corresponding wedge-shape RNFL defect at the location of DH (Fig 2 and Fig 3).

**Fig 4:**
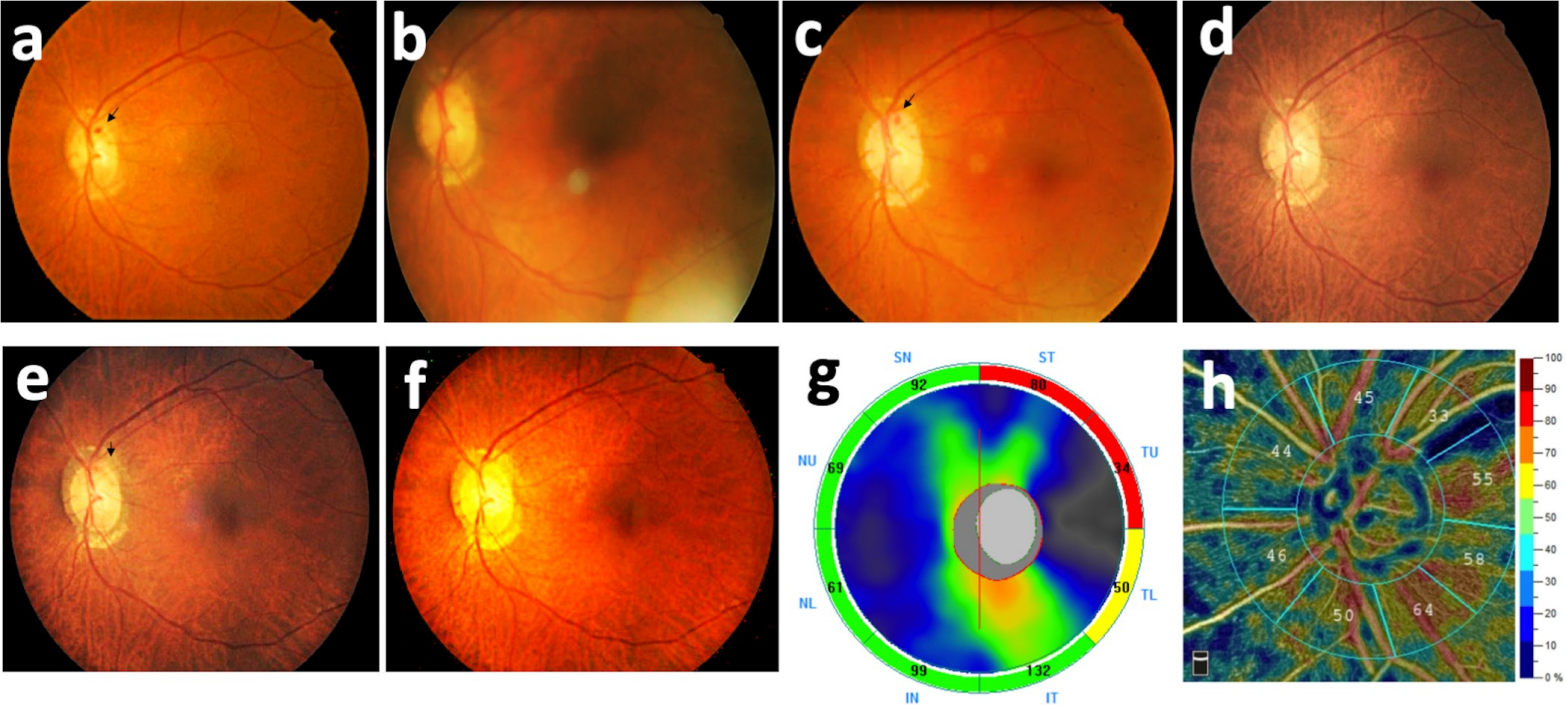
A 68-year-old woman with recurrent DH after a 7-year follow-up. Two episodes of recurrent DH were noted in the same area. (a) Initial DH (arrow). (b) Resolution of initial DH. (c) First recurrent DH (arrow) developed 2 years after the initial DH. (d) Resolution of the first recurrent DH. (e) Second recurrence of DH (arrow), 2 years after the first recurrence. (f) Resolution of the second recurrent DH. (g) & (h) In final visit, optical coherence tomography and angiography revealed RNFL thinning and decreased VD in the superotemporal area corresponding to findings on a color fundus photo.

In our study, five eyes (31.3%) were found to have recurrent DH, out of which three recurrences were observed at the same location with initial DH, whereas the remaining two recurrences were observed at different locations. Among five eyes with recurrent DH, four eyes had one episode of recurrent DH, and one eye developed two episodes of recurrent DH, which was found at the same location with initial DH (Fig 4). DH recurrence was observed at an average of 31 months after initial DH.

A total of 11 eyes (68.8%) presented progression of visual field defect corresponding with the location of DH. Among these cases, the visual field examination demonstrated a mean decrease of 0.64 ± 0.57 dB/year in the mean deviation (MD). The VF examination showed a progressive arcuate scotoma corresponding and extending along the retinal nerve bundle from the location of DH. The change in VF deterioration (dB/year) was significantly poorer than the initial VF test in comparison with the control group (p = 0.008, independent sample t test). In total, 11 eyes (68.8%) had been treated with antiglaucoma agents, despite the mean baseline IOP in these eyes being 14.7 mmHg.

Compared with non-DH eyes, disc scan by OCT-A revealed significantly decreased VD at the sectoral area of the DH (39.38 ± 10.19% vs 50.53 ± 5.84%, p = 0.003) after a long-time follow-up. The mean sectoral RNFL thickness (89.44 ± 26.01 um) was found to be lower than that of the control group (104.62 ± 29.15 um), although the difference was statistically nonsignificant (p = 0.130). As for the whole view, there was a nonsignificant decrease in the peripapillary capillary VD and RNFL thickness (p = 0.287, 0.373). With respect to the macula scan, superficial and deep perifoveal VD were 40.33% and 49.31%, respectively, and were similar to the control group (41.99% and 48.99%, respectively) (p = 0.387 and p = 0.835). Average GCC was 86.13 μm in the DH eye, which was lower than that in the control eye (91.63 μm); however, the difference was statistically nonsignificant (p = 0.190) (Table 2).

**Table 2:** Comparison of structural and functional parameters of nonglaucomatous disc hemorrhages with the fellow eyes.

Variables	DH group	Control group	P value*
**Cup-to-disc ratio**	0.34 ± 0.06	0.31 ± 0.06	0.149
Mean ± SD			
**Initial IOP**(mmHg)	14.42 ± 3.29	15.61 ± 2.81	0.284
Mean ± SD			
**Final IOP** (mmHg)	15.14 ± 2.53	14.92 ± 3.12	0.829
Mean ± SD			
**Average RNFL thickness** (μm)	85.31 ± 12.47	89.81 ± 15.52	0.373
Mean ± SD			
**Sectoral RNFL thickness at DH site** (μm)	89.44 ± 26.01	104.63 ± 29.15	0.130
Mean ± SD			
**GCC**(μm)	86.13 ± 9.51	91.63 ± 13.35	0.190
Mean ± SD			
**Whole image disc VD** (%)	45.58 ± 3.09	46.80 ± 3.51	0.321
Mean ± SD			
**Peripapillary RPC VD (%)**	48.39 ± 3.40	49.26 ± 4.47	0.552
Mean ± SD			
**Sectoral RPC VD at DH site** (%)	39.38 ± 10.19	50.53 ± 5.84	**0.001**
Mean ± SD			
**Macular superficial VD**(%)	40.33 ± 5.15	41.99 ± 4.80	0.387
Mean ± SD			
**Macular deep VD**(%)	49.31 ± 4.03	48.99 ± 3.90	0.835
Mean ± SD			
**VF MD change** (dB/year)	-0.64 ± 0.57	-0.03 ± 0.55	**0.008**
Mean ± SD			

*Compare values between two groups through the independent sample t test

IOP: intraocular pressure, RNFL: retinal nerve fiber layer, DH: disc hemorrhage, GCC: ganglion cell complex, VD: vessel density, RPC: radial peripapillary capillary network, VF MD: visual field mean deviation

To summarize the parameters of OCT-A, DH of nonglaucomatous eyes achieved the capillary dropout of sectoral peripapillary VD compared with fellow eyes after a long-term follow-up. However, measurement of the whole peripapillary VD, superficial and deep macular VD, and whole and sectoral RNFL did not present a statistically significant difference.

## Discussion

In clinical practice, some eyes with DH were neither previously diagnosed with glaucoma nor developed glaucoma after a long-term follow-up. Several studies have reported the relationship between DH and the glaucomatous process.[15, 16] However, a study revealed that 70% of DH was found in eyes without signs of glaucoma.[17] Previous studies have supported that DH can be found in a small percentage of nonglaucomatous eyes (0%–0.4%),[7–9] which was associated with posterior vitreous detachment,[18–20] diabetic papillopathy,[21] ischemic optic neuropathy,[22] optic disc drusen, vascular occlusive diseases of the retina, leukemia, and systemic lupus erythematosus.[23]

In the current study, all eyes presented with a peripapillary hemorrhage. It is the most common type of DH that involves both the neuroretinal rim and parapapillary area.[24] This type of DH characteristically presents with a splinter-shaped superficial hemorrhage, whereas the other type, cup hemorrhage, is characterized by deeply located round and blotchy appearance. Cup hemorrhage was not found in our study. Peripapillary hemorrhage has been reported to be associated with a localized RNFL defect, whereas the location of a cup hemorrhage is not correlated with the direction of the RNFL defect.[24] The present study found that the inferotemporal quadrant was the most common site for both initial and recurrent DH; this finding is compatible with that of a previous study.[20]

A study proposed a relationship between DH and the rapid rate of structural and functional progression.[3] However, the mechanism of DH remained unclear. Vascular abnormality has been proposed as a cause of DH.[10, 25] However, another theory suggested that DH was caused by mechanical vascular disruption at the lamina cribrosa or the margin of the optic disc and RNFL defect.[26, 27] Recent studies have focused on the effect of the focal lamina cribrosa defect on the development of DH.[28, 29]

Prior studies have indicated that localized thinning of the RNFL develops in glaucomatous eyes with DH measured by OCT.[30, 31] Our study revealed a decrease in the localized RNFL thickness at a prior DH site in non-glaucomatous eyes, although it did not achieve significance. Owing to the advent of OCT-A, several studies have reported that decreased choroidal microcirculation was detected in glaucomatous eyes with hemorrhage; DH was correlated with glaucoma progression.[11, 32] However, no study has investigated the change in microcirculation in nonglaucomatous eyes with DH. The present study is the first to reveal that significantly reduced sectoral peripapillary VD is also found in nonglaucomatous eyes with DH in comparison with the control eye after a long-term follow-up. This indicates that spatially compatible peripapillary capillary dropout as a microcirculation defect may precede RNFL defect at a prior DH site in nonglaucomatous eyes.

In summary, a significantly declined sectoral VD with corresponding functional visual field defect at the DH site was found in our study; however, the RNFL defect including the whole view and localized area was not significant in this study. This indicates that the microvasculature change is an earlier sign than the RNFL change at the DH site in these eyes when the visual function deteriorated during the long-term follow-up. OCT-A with measurement of ocular microcirculation may be a superior tool in monitoring nonglaucomatous eyes with DH than merely RNFL measurement by OCT.

We found that the localized pigment formation of the optic disc developed at a prior DH site in two eyes at respectively 2- and 4-year follow-ups in our study. To our knowledge, there is limited literature reporting this clinical feature in DH eyes. As a brief report, Klais and Spaide illustrated submacular placoid pigmentation after macular edema resolution in CRVO after the intervention was due to hyperplasia and hypertrophy of the RPE.[33] Similarly, DH might be associated with retinal inflammation and might induce the migration of the pigment. Pigmentation of the optic disc is uncommon, and secondary optic disc pigmentation may be caused by the accumulation of pigment granules resulting from hemorrhage.[4] We may postulate that the localized pigmentation of the disc margin is a result of DH absorption. Retinal pigmentation of the disc margin with the RNFL defect is noted as a consequence or a clue of DH. More cases should be studied to understand and identify its detailed characteristics.

Our current study also revealed the corresponding visual field defect developing and progressing during a long-term follow-up. Glaucoma treatment intensification may be beneficial in reducing the rate of RNFL thinning in a DH quadrant after DH in glaucomatous eyes.[31] For glaucoma eyes, there was consensus that DH is more of a sign than a risk factor for glaucoma progression.[34] In the present study, glaucoma treatment was administered in eyes with more RNFL defect at the DH site even though the patient was not initially diagnosed with glaucoma. However, it is noteworthy that the progression of the visual field defect could not be prevented even after treatment with antiglaucoma drugs in these eyes (68.8%). Lee et al. illustrated that in NTG, no difference of VF progression was observed for untreated IOP < 15 mmHg and ≥ 15 mmHg groups (0.350 ± 0.028 dB/y and 0.353 ± 0.026 dB/y, respectively). However, our DH eyes presented with more severe VF progression than NTG cases.[35] For most cases receiving glaucoma treatment, we could not compare the progression rate of RNFL and microcirculation defect in eyes receiving and not receiving glaucoma treatment. Additional randomized prospective studies are necessary to prove whether the effect of intensified treatment for nonglaucomatous eyes is beneficial.

Previous studies mostly focused on DH in glaucomatous patients. The present study revealed a significantly declined sectoral VD with corresponding functional visual field defect at the DH site in nonglaucomatous eyes. However, we found no significant sectoral RNFL change at the DH site in these nonglaucomatous eyes compared with fellow eyes. These findings suggest that decreased peripapillary VD may precede RNFL thinning at the DH site in nonglaucomatous eyes while the visual function declines.

Our study has several limitations. Because OCT-A imaging is a newly emerging technique, we performed this examination only after OCT-A was available. Furthermore, OCT-A and visual field examination were not performed routinely because of the nonglaucomatous condition. Other limitations include the retrospective nature of the study and small number of nonglaucomatous eyes with DH. However, the long follow-up period in our study may provide more convincing evidence on the structural and functional effect of DH. Moreover, evaluation of peripapillary VD may provide more detailed characteristics of DH.

## Conclusion

DH is a sign of glaucomatous progression, but it may also present in the normal eye. Therefore, careful diagnosis of glaucoma in fresh eyes with DH is crucial. Decreased peripapillary microvasculature may precede the RNFL defect at a prior DH site in nonglaucomatous eyes. Although it is customary to treat eyes with an RNFL defect in DH eyes associated with VF defect progression, it is unclear whether the treatment will benefit these patients. A randomized trial for effectiveness of the treatment for DH is necessary.
